# Three-Dimensional Hierarchical Reticular Nanostructure of *Fulfora candelaria* Wing Decorated by Ag Nanoislands as Practical SERS-Active Substrates

**DOI:** 10.3390/nano8110905

**Published:** 2018-11-05

**Authors:** Mingli Wang, Yuhong Wang, Xiaoya Yan, Xin Sun, Guochao Shi, Keqin Zhang, Lijian Ren, Wanli Ma

**Affiliations:** 1 State Key Laboratory of Metastable Materials Science & Technology and Key Laboratory for Microstructural Material Physics of Hebei Province, School of Science, Yanshan University, Qinhuangdao 066004, China; wangyh@stumail.ysu.edu.cn (Y.W.); yxy521hp@stumail.ysu.edu.cn (X.Y.); sunxin@ysu.edu.cn (X.S.); sgc@stumail.ysu.edu.cn (G.S.); 2 Hebei Huadian Guyuan Wind Power Co., Ltd., Zhangjiakou 075000, China; chenbin@chd.com.cn; 3 Department of Mathematics, North Carolina State University, Raleigh, NC 276968205, USA; wanlimaphil@gmail.com

**Keywords:** surface-enhanced Raman scattering, three-dimensional finite-difference time-domain simulation, *Fulfora candelaria* wing, recognition ability, practicability

## Abstract

Although surface-enhanced Raman scattering (SERS) technology has been widely explored nowadays in various fields, the fabrication of practical SERS-active substrates with prominent recognition ability for various analyte molecules is still defective. Natural *Fulfora candelaria* wing (FCW) with three-dimensional (3D) hierarchical reticular nanostructure was selected as a new bioscaffold for rough silver (Ag) nanoislands to be assembled on to prepare a practical SERS substrate (Ag/FCW substrate). By adjusting the sputtering time of metal Ag, the morphology of the substrates could be easily tuned to control the formation and distribution of “hot spots”. Three-dimensional finite-difference time-domain (3D-FDTD) simulation indicated that the excellent SERS performance under optimal morphology was ascribed to the local enhanced electric field in rough Ag surface and effective “hot spot” areas. The SERS measurement results show that the optimal Ag/FCW substrates had high SERS performance in terms of Raman signal sensitivity, reproducibility, uniformity and recognition ability for various analyte molecules. Coupled with flexibility of the biological substrates and the cost effectiveness, the sensitive SERS detection of varied analytes based on Ag/FCW substrates offered great potential for practical applications.

## 1. Introduction

Surface-enhanced Raman scattering (SERS) has attracted considerable interest owing to its application as a potent tool for the rapid and sensitive analysis of chemical and biological molecules by giving real-time molecular vibrational information [1]. When the interaction of pyridine molecules and the surface of a roughened silver electrode led to a significant increase in Raman signals, the SERS phenomenon was first observed in 1974 [2]. As is well known, the coherent wave oscillations of metallic surface-electrons will be excited by incident laser, resulting in the amplification of electromagnetic (EM) fields at nanometric roughened surface, and thus effectively increase the Raman scattering cross-sections of analyte molecules adsorbed or located on and near the metallic surface [2,3]. Therefore, the EM enhancement mechanism of SERS is attributed to the localized surface plasmon resonance (LSPR). Meanwhile, Raman scatterers located at the interparticle junctions present strong SERS enhancement, exceeding that of the isolated nanoparticles by several orders of magnitude [4], and thus the Raman signals can be enhanced greatly when target molecules locate in the gaps between neighboring metal nanoscaled units (called “hot spots”). These “hot spots”, which can strongly increase the intensity of the local electric field around them, enable the SERS detection of extremely low concentration or even single molecule [5,6]. In addition, charge transfer (CT) enhancement is another widely accepted enhancement mechanism induced by an amplification of molecule polarization formed by the interaction between the molecule and metallic surface, presenting 10–10^2^ enhancement, while the enhancement of EM mechanism can reach 10^4^–10^12^ [7,8].

Size, geometry and inter-particle separation of metallic particles are crucial to efficiently tune the SERS performance of the substrates [9]. In recent years, scientists have made significant efforts to design SERS-active substrates in various geometries including nanopillars [10,11,12], nanocap [7], nanostars [13,14], nanotree [15], nanosheet [16] and nanorods [1,17]. Although certain advances have been achieved, which obtain a series of SERS substrates with some special advantages, it remains difficult for these substrates to simultaneously possess high-sensitivity, outstanding reproducibility, prominent uniformity and excellent recognition performance for various molecules. Three-dimensional (3D) natural biological structures with nanoscale arrays as templates are adopted to demonstrate the correlation between morphology and SERS activity of the substrates, which arouses great attention recently [18,19,20]. An interesting morphology of a 3D hierarchical reticular structure is discovered on the wings of *Fulfora candelaria*, the use of which as the bioscaffold can not only offer large surface area for the deposition of metallic nanoparticles and the absorption of analyte molecules, but also obtain the full utilization of incident laser, which are the prominent advantages that one-dimensional (1D) and two-dimensional (2D) SERS substrates do not have [21]. In addition, the natural *Fulfora candelaria* wing (FCW) directly acts as the substrate template, which greatly simplifies the complex experimental operations during the process of template fabrication and avoids the environmental pollution caused by the experimental garbage. Importantly, it effectively avoids the uncertainty in synthesis. Compared with gold (Au) and copper (Cu), the plasmonic spectral window covered by silver (Ag) nanostructures in the entire range from visible to infrared and Ag exhibits the greatest enhancement among all of the metallic materials [22,23]. Therefore, the combination of *Fulfora candelaria* wings and Ag is explored in our experiments.

In this paper, we describe the experiments of a cost-effective, high-sensitive and practical 3D SERS-active substrate (Ag/FCW substrate) based on the *Fulfora candelaria* wing with hierarchical reticular nanostructure and rough Ag nanoislands fabricated by magnetron sputtering technology. A strong substrate morphology-dependent trend in the measurement of SERS signals was observed to obtain an optimal SERS-active substrate that gave the highest SERS sensitivity and the Ag/FCW substrate with a 10 min Ag sputtering time (Ag/FCW-10 substrate) stood out. The abundant effective “hot spots” distributed in the neighboring Ag nanoislands and the nanosized roughness on the surface of the Ag/FCW-10 substrate made this SERS-active substrate possess potential local field enhancement effect to analyte molecules. Furthermore, the SERS behaviors of Ag/FCW-10 substrates were investigated by SERS measurements under various probe molecules, and displayed high-sensitivity, outstanding reproducibility, prominent uniformity and excellent molecular recognition performance. In addition, the Ag/FCW-10 substrates based on *Fulfora candelaria* wings as the bioscaffold were flexible, hence the integrality of the SERS substrates would not be influenced if the substrates were bent to a certain extent, and this trait would be crucial to situ detection in practical applications. Taken altogether, Ag/FCW-10 substrates with many prominent advantages can serve as high performance and efficient SERS platforms for practical applications.

## 2. Experiments and Methods

### 2.1. Materials and Instruments

Rhodamine 6G (R6G, C_28_H_31_N_2_O_3_Cl), 4-aminothiophenol (4-ATP, C_6_H_7_NS), cypermethrin (CYP, C_22_H_19_C_l2_NO_3_) and ethyl alcohol absolute (C_2_H_6_O) were purchased from J&K Scientific LTD (Beijing, China). Crystal violet (CV, C_25_H_30_ClN_3_) was obtained from Tianjin Kemiou Chemical Reagent Co., Ltd. (Tianjin, China). In addition, deionized water was acquired from Key Laboratory for Microstructural Material Physics of Hebei Province, which was used throughout the whole experiment unless otherwise indicated. *Fulfora candelaria* wings (bought from Beijing Jiaying Grand Life Sciences Co., Ltd., Beijing, China) were used as natural substrate templates to be decorated by Ag nanoislands with radio frequency (RF) magnetron sputtering apparatus (DHRM-3, Hangzhou Dahua Apparatus Manutacture Co., Ltd., Hangzhou, China). Raman measurements were performed using confocal Raman system (inVia) with an extended model and the substrate morphology was described with the scanning electron microscope (SEM, Hitachi S-4800 II, Hitachi Ltd., Tokyo, Japan). The UV-vis absorption spectra were monitored by UV-2550 UV-vis spectrophotometer (Shimadzu, Shanghai, China).

### 2.2. Fabrication of SERS-Active Substrates

**Figure 1:**
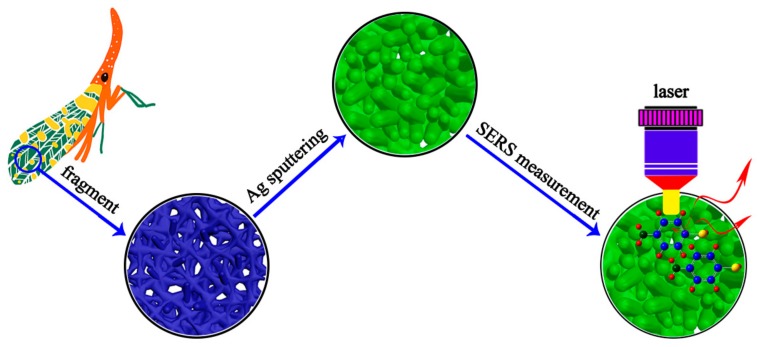
Schematic illustration of the fabrication program of the practical Ag/FCW substrates and SERS measurement.

The procedures for fabricating Ag decorated *Fulfora candelaria* wing as a high-sensitive, cost-effective and practical SERS-active substrate are schematically depicted in Figure 1. Briefly, the *Fulfora candelaria* wings were cleaned in ethyl alcohol to remove the stain and dried naturally. Before the Ag sputtering process, the wings were fixed onto glass slides. Subsequently, Ag nanoislands were decorated onto the prepared wings by RF magnetron sputtering technology. In this process, the electric current was maintained at 170 mA; the voltage was controlled to 90 V; and the vacuum degree was 10^−3^ Pa. To explore the influence of the substrate’s morphology on the SERS performance, the sputtering time was set to 5, 10, 15, 20, 25 and 30 min, and the as-fabricated substrates were labeled as Ag/FCW-5, Ag/FCW-10, Ag/FCW-15, Ag/FCW-20, Ag/FCW-25 and Ag/FCW-30, respectively. To slow down the oxidation of Ag’s surface, all of the SERS-active substrates were stored in a vacuum environment.

### 2.3. SERS Measurements

The 10^−6^ M R6G solution was dropped onto different substrates fabricated by changing the Ag sputtering time. In view of the familiar vibration modes of R6G molecules, it is easy to compare their signal intensities further to pick out the optimal substrate with a more suitable sputtering time. Subsequently, 10^−4^ M CV alcoholic solution was prepared and diluted to various concentrations ranging from 10^−5^ M to 10^−10^ M. Then, these solutions were dropped onto the optimal Ag/FCW substrates, respectively, and the solutions were allowed to evaporate under a vacuum condition. The 4-ATP solution with a concentration range from 10^−3^ M to 10^−11^ M and the CYP solution with a concentration range from 10^−3^ g/L to 10^−10^ g/L were all prepared. After that, the optimal Ag/FCW substrates were immersed into the 4-ATP solutions with different concentrations for 2 h. This operation was also performed in CYP solution. Next, the treated substrates were cleaned in ethyl alcohol absolute to remove the unattached solutions, and then dried in nitrogen to ensure that a complete self-assembled monolayer was formed on the substrate surface. Finally, the Raman system was adopted to directly measure the SERS performance of these treated Ag/FCW substrates. The incident laser of 532 nm with a power of 0.1 mW was selected in our experiment. In addition, each Raman spectrum was recorded for 10 s at the spectral resolution of 1 cm^−1^.

## 3. Results and Discussion

### 3.1. Characterization and Three-Dimensional Finite-Difference Time-Domain Simulation

**Figure 2:**
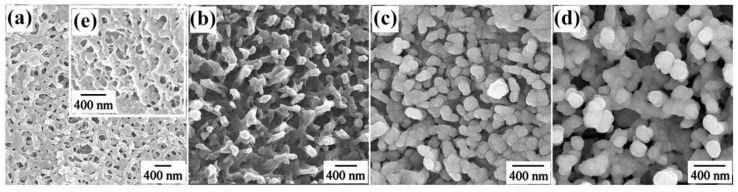
Typical top-view SEM images of the Ag/FCW substrates with different Ag sputtering times: (**a**) 0 min; (**b**) 5 min; (**c**) 10 min and (**d**) 15 min. (**e**) Side-view SEM image of the natural *Fulfora candelaria* wing.

The surface of natural *Fulfora candelaria* wing presented an interesting morphology that the hierarchical reticular nanostructure with obvious branches and cavities was exhibited, as shown in Figure 2a. Especially, in the side-view SEM image, as displayed in Figure 2e, tridimensional irregular branch arrays could be observed. As is well known, the 3D SERS-active substrates can not only provide abundant “hot spots” which play an important role in producing strong local field enhancement, but also provide large surface area for the deposition of metallic nanoparticles and the absorption of analyte molecules. Hence, the *Fulfora candelaria* wing was used as a bioscaffold for the fabrication of SERS-active substrates in our experiment. At a short sputtering time of 5 min (Figure 2b), the net was coated with Ag nanoislands, which led to a decrease of the cavity diameter, and rough nanopillars were highlighted. With the increase of sputtering time, the cavities were gradually filled with nanoislands, resulting in the cavities disappearing completely. Specifically, when the sputtering time reached 10 min, as exhibited in Figure 2c, not only were the cavities replaced by dense nanopillars, but also abundant nanogaps between neighboring Ag nanoislands were displayed, which would supply a giant local field enhancement. Furthermore, the nanosized roughness on the surface of the Ag nanoislands would also present potential enhancement effect to a certain extent. More importantly, the rough surface of nanoislands effectively increased the attachment area to absorb probe molecules under the unit laser illumination area. Sputtering for 15 min, some adjacent nanopillars were connected by the constantly sputtering Ag nanoislands and formed a whole, plus the stacking of several nanoislands, making the nanogaps between neighboring nanoislands obviously become larger, as displayed in Figure 2d.

**Figure 3:**
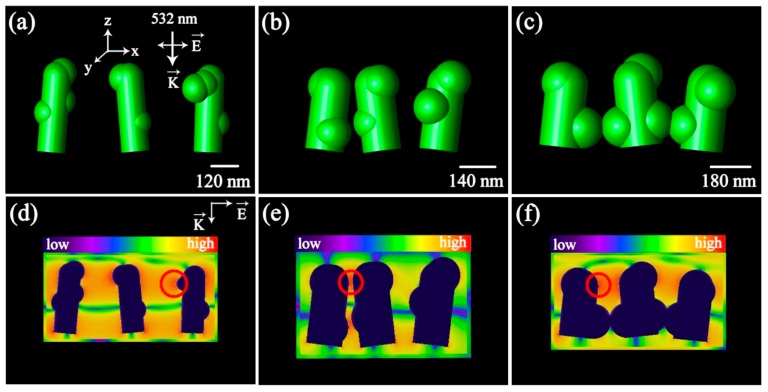
(**a**–**c**) The 3D-FDTD models of Ag/FCW-5, Ag/FCW-10 and Ag/FCW-15, respectively; and (**d**–**f**) x-z views of the electric field distribution around corresponding substrates in (**a**–**c**).

According to the observation of substrate’s morphology in the SEM images, the models of corresponding substrates with different roughness and nanogaps were built, as shown in Figure 3a–c. The electric field distributions for each morphology of Ag/FCW substrates using three-dimensional finite-difference time-domain (3D-FDTD) analysis under a rectangular-shaped continuous laser with a wavelength of 532 nm were calculated, which could give a fundamental understanding for the outstanding SERS sensitivity of the Ag/FCW substrates. To investigate the enhancement behavior, the x-z views of the field distributions according to different substrates are exhibited in Figure 3d–f, in which the laser was shot along the *K* direction and the polarization direction was *E*. The highest calculated local EM enhancement factor (1.19 × 10^5^) was given to the Ag/FCW-10 substrates corresponding to the Ag/FCW substrate model exhibited in Figure 3b, which was theoretically predicted by |*E*(*ω*)/*E_inc_*(*ω*)|^4^ [19,24]. Because of the complexity of the real nanostructure of the Ag/FCW substrates, the model compared the effects of different roughness and nanogaps on SERS performance, which could only explain part of the enhancement mechanism, and thus there might be a deviation from the real results. In Figure 3d–f, it can be seen that strong field enhancement was displayed on both the surface of the Ag nanoislands and in the nanogaps between two neighboring nanoislands. Specifically, the nanogaps in Ag/FCW-10 substrates played the most important role in introducing strong electrical field, as shown in Figure 3e, where local electrical field were effectively amplified, while other substrates mainly produced enhancement effect by the rough surface of Ag nanoislands. These results demonstrate the influence of substrate’s morphology on SERS performance and also indicate that the effective “hot spots” were the main reason for high SERS sensitivity and strong EM enhancement in detecting analytical molecules.

### 3.2. SERS Performances

**Figure 4:**
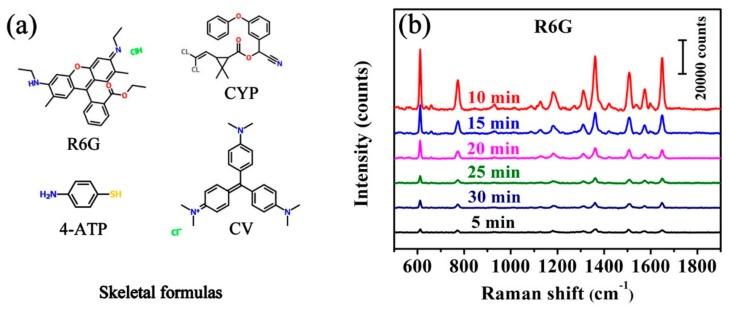
(**a**) The Skeletal formulas of the probe molecules used in this study; and (**b**) SERS spectra of 10^−6^ M R6G alcoholic solution absorbed onto corresponding substrates prepared at different Ag sputtering times (from 5 min to 30 min).

To verify the practical value of the Ag/FCW SERS-active substrates, R6G, CV, 4-ATP and CYP were selected as probe molecules to estimate their SERS performance. The Skeletal formulas of all tested molecules are shown in Figure 4a. R6G is one of the dyes with well-established vibrational features. CV is a dose-related carcinogen which can lead to liver cancer, certain tumors and sarcomas in rodents. 4-ATP is an intermediate for pesticides, medicines and dyes, and can be effectively absorbed onto the SERS substrates [25]. CYP is an insecticide used to protect the crops from insect pests, however, its residues can pose significant health risks to human when applied improperly [26]. To screen out the optimal substrates from the as-prepared Ag/FCW substrates, we measured the Raman signal as a function of 10^−6^ M R6G solution on Ag/FCW substrates with different sputtering times to observe their SERS behaviors, as shown in Figure 4b. The Raman characteristic bands of R6G at 611, 773 and 1182 cm^−1^ were associated with C–C–C ring in-place, C–H out-of-place and C–H in-place bending, respectively. The other features at 1310, 1362, 1509, 1574 and 1650 cm^−1^ were all assigned to aromatic C–C stretching vibrations [15,27]. Compared with the Ag/FCW substrates which exhibited low signal intensities, the Ag/FCW-10 substrates promoted the Raman intensity displaying the best SERS performance in terms of the enhancement effect.

**Figure 5:**
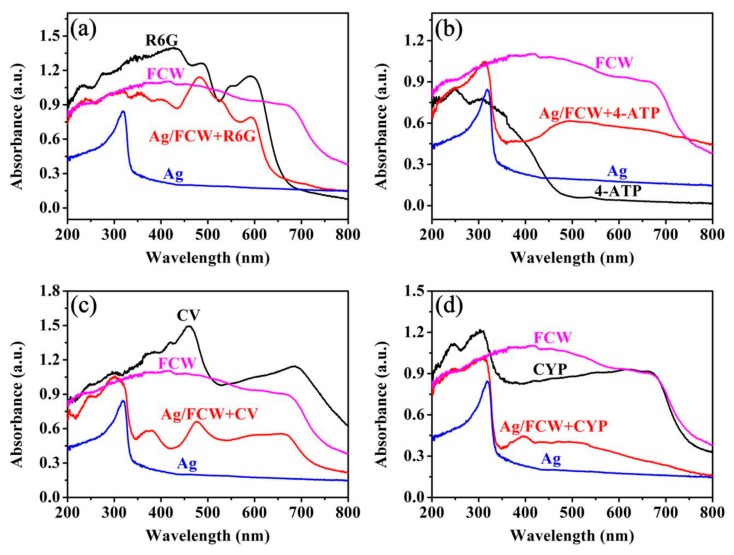
The comparison of UV-vis absorption spectra of: (**a**) R6G, Ag, *Fulfora candelaria* wing (FCW) and Ag/FCW-10 substrates absorbed with R6G molecules; (**b**) 4-ATP, Ag, FCW and Ag/FCW-10 substrates absorbed with 4-ATP molecules; (**c**) CV, Ag, FCW and Ag/FCW-10 substrates absorbed with CV molecules; and (**d**) CYP, Ag, FCW and Ag/FCW-10 substrates absorbed with CYP molecules.

**Figure 6:**
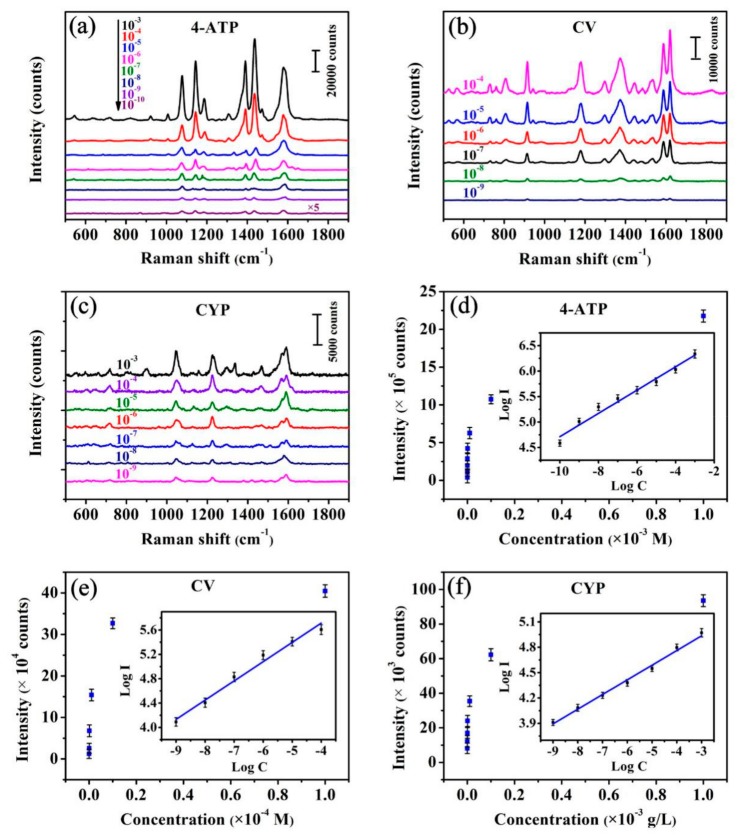
SERS spectra obtained from different concentrations of: (**a**) 4-ATP; (**b**) CV; and (**c**) CYP solutions absorbed on the Ag/FCW-10 substrates. The quantitative relation curves for the logarithm of the integrated SERS intensities and corresponding solution concentrations of: (**d**) 4-ATP; (**e**) CV; and (**f**) CYP.

The UV-vis absorption spectra of R6G, 4-ATP, CV, CYP, Ag, *Fulfora candelaria* wing and the Ag/FCW-10 substrates absorbed with each of analytes were all measured and the correlation spectra were compared, as exhibited in Figure 5. In Figure 5a, compared with the absorption peak of Ag at around 318 nm, several absorption peaks belonging to R6G appeared obviously after adding R6G to the Ag/FCW-10 substrates, while the *Fulfora candelaria* wing had little effect on the absorption spectrum. The same experimental phenomena took place in 4-ATP, CV and CYP. Therefore, the use of 532 nm laser as the excitation source in the Raman system to obtain all of the Raman signals in our experiment could not only enchanced the local electromagnetic field at Ag surface where the interaction of the incident laser with the electrons occurred, but also achieved the resonance of analyte molecules and incident laser, contributing to improve the detection limit of analytes. The spectra of 4-ATP, CV and CYP with a changed concentration on the optimal Ag/FCW substrates were recorded, as shown in Figure 6a–c, respectively. As expected, the lower were the concentrations, the weaker were the signal intensities. However, when the concentration reached a low concentration of 10^−10^ M for 4-ATP, it still exhibited distinct Raman characteristic peaks, as displayed in Figure 6a. The obvious enhancements of signal intensities at 1143, 1391 and 1436 cm^−1^ were attributed to the appearance of 4,4’-dimercaptoazobenzene (DMAB), which was transformed from 4-ATP induced by the high-power laser [28,29]. The SERS spectra of CV with different concentrations absorbed onto the optimal substrates were also measured to test the sensitivity, as shown in Figure 6b. The Raman bands at 1178, 1372 and 1586 cm^−1^ were associated with C–H, C–N and aromatic C–C stretching vibrations, respectively. The other characteristic bands at 729, 806 and 915 cm^−1^ were assigned as C–N–C symmetric stretch of the dimethylamino groups [30]. The main vibrational features of CV could be identified clearly at the concentration of 10^−9^ M. The high sensitivity of the Ag/FCW-10 substrates was also reflected in the low detection concentration of 10^−9^ g/L to CYP, as displayed in Figure 6c. Such low detection limits could be related to the enhanced local EM field induced by the plasmonic coupling of rough Ag nanoislands and the resonant Raman effect. To further investigate the SERS behaviors of 4-ATP that absorbed onto the Ag/FCW-10 surface and irradiated by a high-power laser, the changes of the Raman intensities at 1581 cm^−1^ corresponding to the different spectra in Figure 6a as a function of the concentrations were plotted in log scale. As displayed in Figure 6d, a good linear SERS response with a high confidence coefficient (R = 0.973) for the peaks at 1581 cm^−1^ between the logarithmic signal intensities and the logarithmic concentrations was obtained. Similarly, the logarithms of the integrated SERS intensities and corresponding solution concentrations of CV and CYP all possessed great linear relationships, as exhibited in Figure 6e,f. High confidence coefficients of 0.980 and 0.991 for CV and CYP, respectively, were calculated. The linear response over the concentration range of each detection solution provides a simple platform for implementing the determination of the unknown concentration of this solution with Ag/FCW-10 substrates.

**Figure 7:**
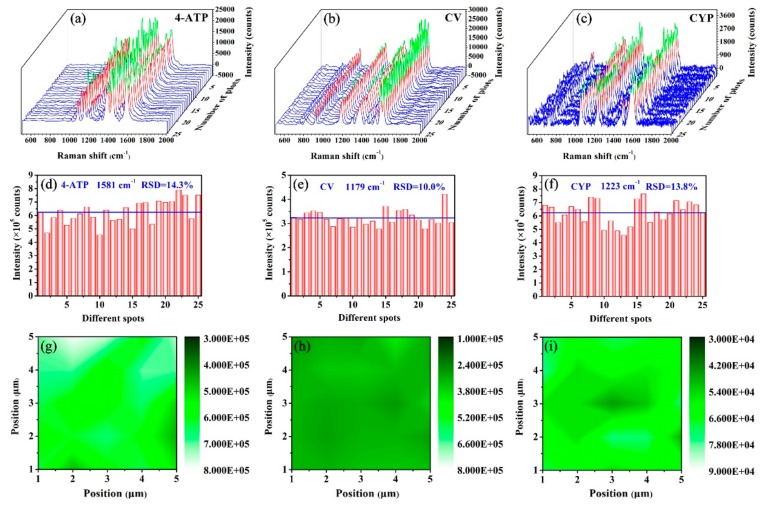
SERS spectra of: (**a**) 10^−5^ M 4-ATP solution; (**b**) 10^−5^ M CV solution; and (**c**) 10^−4^ g/L CYP solution obtained at 25 spots randomly chosen from 5 Ag/FCW-10 substrates. (**d**–**f**) The corresponding intensity distribution of 4-ATP at 1581 cm^−1^, CV at 1179 cm^−1^ and CYP at 1223 cm^−1^ in (**a**–**c**) (the average intensities are marked with blue lines). SERS mapping of the peak across a 5 × 5 μm^2^ area measured at: (**g**) 1581 cm^−1^ for 4-ATP; (**h**) 1179 cm^−1^ for CV; and (**i**) 1223 cm^−1^ for CYP.

The substrate-to-substrate reproducibility was one of the most important factors in verifying the practicability of the SERS substrates, which was demonstrated by the spectra of 10^−5^ M 4-ATP solution, 10^−5^ M CV solution and 10^−4^ g/L CYP solution recorded on 25 randomly selected spots of five prepared Ag/FCW-10 substrates. As exhibited in Figure 7a, the signal intensities of main characteristic peaks for 4-ATP absorbed onto the Ag/FCW-10 surface and irradiated by a high-power laser were consistent to a certain extent, and the value of the relative standard deviation (RSD) for the vibration at 1581 cm^−1^ was calculated as 14.3%, as shown in Figure 7d. A further illustration was performed by collecting the SERS spectra of CV and CYP at 25 different spots, as shown in Figure 7b,c. As exhibited in Figure 7e,f, the values of RSD were estimated to be 10.0% and 13.8% for CV and CYP at 1179 cm^−1^ and 1223 cm^−1^, respectively. In addition, a 5 × 5 μm^2^ area with a step-size of 1 μm in a random Ag/FCW-10 substrate was selected to detect the SERS behaviors under different probe molecules, and the results are displayed in Figure 7g–i. In the SERS mappings, each pixel represents a corresponding peak intensity, thus the relatively uniform color distribution illustrates the point-to-point reproducibility of our substrates. The high-sensitivity, outstanding reproducibility, prominent uniformity and the excellent recognition performance for various molecules further suggest the practical value of the Ag/FCW-10 substrates.

### 3.3. EF Calculation

**Figure 8:**
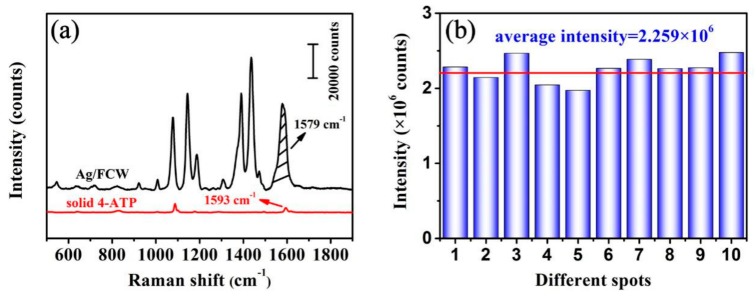
(**a**) SERS spectrum of 10^−3^ M 4-ATP solution absorbed onto the Ag/FCW-10 substrates and Raman spectrum of solid 4-ATP; and (**b**) the intensity distribution of 10^−3^ M 4-ATP at 1579 cm^−1^ measured from 10 different pots (the average intensity is marked with red line).

The SERS spectrum of 10^−3^ M 4-ATP on Ag nanoislands prepared by sputtering the *Fulfora candelaria* wing with Ag for 10 min and the normal Raman spectrum of solid 4-ATP are contrasted in Figure 8a. Because DMAB could also give the Raman characteristic peak of 1593 cm^−1^ at similar position to that of 4-ATP with an essentially constant integrated peak intensity [29], the peak band at 1593 cm^−1^ in the SERS spectrum of 4-ATP solution absorbed onto the Ag/FCW-10 substrate was selected to approximately calculate the enhancement factor (EF) of this substrate, which was mainly for the purpose of comparing with the similar SERS research work done by colleagues. Obviously, the Raman characteristic peak at 1593 cm^−1^ in Raman spectrum was shifted to 1579 cm^−1^ in SERS spectrum, which was due to the direct interaction of -SH group in 4-ATP with Ag surface to form a strong Ag–S bond [31]. Moreover, the improved signal intensities of 10^−3^ M 4-ATP at 1579 cm^−1^ with an average intensity of 2.259 × 10^6^ collected from the Ag/FCW-10 substrates were recorded (Figure 8b). The solid 4-ATP sample was used as a reference substrate to estimate the SERS analytical EF of Ag/FCW-10 substrates and the average EF could be calculated using the following formula [32]:

EF = (*I*_surf_/*I*_bulk_) × (*N*_bulk_/*N*_surf_) (1)

where *I*_surf_ and *I*_bulk_ are the average signal intensities of vibration at 1579 cm^−1^ in SERS spectra and 1593 cm^−1^ in Raman spectra, respectively, which could be obtained by experiment, and thus the ratio *I*_surf_/*I*_bulk_ was calculated to be ~66.08 (~2.26 × 10^6^/~3.42 × 10^4^). In addition, *N*_bulk_ and *N*_surf_ represent the number of 4-ATP molecules in the bulk solid sample and the surface of Ag/FCW-10 substrates, respectively. For solid 4-ATP sample, the value of *N*_bulk_ could be estimated by the accepted formula [32]:

*N*_bulk_ = *V* × *ρ* × *N*_A_/*M* (2)

where *ρ* is the density of 4-ATP (1.18 g/cm^3^), *M* represents the molecular weight (125.19 g/mol) and *N*_A_ is Avogadro’s number (~6.022 × 10^23^). Moreover, the variable *V* is the collection volume of the solid sample monitor, which was calculated to be ~7.85 μm^3^ under the illumination area diameter of ~1 μm and the penetration depth of 10 μm in the measured Raman system. Further, the value of *N*_bulk_ was estimated to be ~4.46 × 10^10^. *N*_surf_ is the number of 4-ATP molecules absorbed onto the surface of the Ag/FCW-10 substrate, where the 4-ATP molecules were distributed in a monolayer. Considering the surface morphology of Ag/FCW-10 substrates, the illumination area was calculated to be ~1.57 μm^2^. Given that the surface area of one 4-ATP molecule was ~0.2 nm^2^ [25,33], the value of *N*_serf_ was estimated to be ~7.85 × 10^6^. Therefore, the ratio *N*_bulk_/*N*_serf_ of ~5.68 × 10^3^ (4.46 × 10^10^/7.85 × 10^6^) was obtained. As a result, the EF for Ag/FCW-10 substrates was calculated to be ~3.75 × 10^5^.

## 4. Conclusions

In summary, we have presented a practical strategy for decorating Ag nanoislands onto natural *Fulfora candelaria* wings as SERS-active substrates, where the introduction of *Fulfora candelaria* wing with 3D hierarchical reticular structure as a bioscaffold made the preparation process greatly simplified, while obtaining the high SERS performance. The morphology of the Ag/FCW substrates could be simply tuned by adjusting the Ag sputtering time and the SERS measurement results indicated that the Ag/FCW-10 substrates with abundant effective “hot spots” exhibited best SERS performance in terms of the enhancement effect. Furthermore, the Ag/FCW-10 substrates with high sensitivity (a low detection limit of 10^−10^ M for 4-ATP, 10^−9^ M for CV, and 10^−9^ g/L for CYP), excellent reproducibility (RSD < 15%), outstanding uniformity, unique recognition ability for various molecules (R6G, 4-ATP, CV and CYP), prominent flexibility and great cost effectiveness could serve as a high-performance SERS platform for in situ determination and create promising detection as a functional component in practical applications.
